# The efficacy and safety of P2Y12 inhibitor monotherapy in patients after percutaneous coronary intervention

**DOI:** 10.1002/clc.23305

**Published:** 2019-11-28

**Authors:** Liman Luo, Menglu Fu, Yuanyuan Li, Zhihui Chen, Jing Yu, Jinlan Luo, Shuiqing Hu, Ling Tu, Xizhen Xu

**Affiliations:** ^1^ Department of Geriatric Medicine, Tongji Hospital, Tongji Medical College Huazhong University of Science and Technology Wuhan China; ^2^ Division of Cardiology, Department of Internal Medicine, Tongji Hospital, Tongji Medical College Huazhong University of Science and Technology Wuhan China; ^3^ Hubei Key Laboratory of Genetics and Molecular Mechanisms of Cardiological Disorders Wuhan China

**Keywords:** meta‐analysis, P2Y12 inhibitor monotherapy, percutaneous coronary intervention

## Abstract

The optimal antiplatelet therapy after percutaneous coronary intervention (PCI) remains to be elucidated. Monotherapy with a P2Y12 inhibitor may be inferior to dual antiplatelet therapy in patients after PCI. PubMed, EMBASE (by Ovidsp), Web of Science, and The Cochrane Library were searched from database inception to 2 October 2019. The composite of cardiovascular outcomes, all‐cause mortality, myocardial infarction (MI), stroke, stent thrombosis, and major bleeding were evaluated. Pooled outcomes were presented as relative risk (RR) and 95% confidence intervals (CIs). A total of four trials randomizing 29 089 participants were included. Compared with the dual antiplatelet therapy group (n = 14 559), the P2Y12 inhibitor monotherapy group (n = 14 530) significantly decreased the incidence of bleeding events (2.0% vs 3.1%; RR: 0.60; 95% CI: 0.43‐0.84; *P* = .005). There were no significant differences in all‐cause mortality (1.3% vs 1.5%; RR: 0.87; 95% CI, 0.71‐1.06; *P* = .16), myocardial infarction (2.1% vs 1.9%; RR, 1.06; 95% CI, 0.90‐1.25; *P* = .46), stroke (0.6% vs 0.5%; RR, 1.18; 95% CI, 0.67‐2.07; *P* = .57), or stent thrombosis (0.5% vs 0.4%; RR, 1.14; 95% CI, 0.81‐1.61; *P* = .44) between the two groups. P2Y12 inhibitor monotherapy did not show any significant difference in the adverse cardiac and cerebrovascular events, but markedly decreased the risk of bleeding among patients after PCI vs dual antiplatelet therapy. However, it still needs to be further confirmed due to limited data.

## 1 INTRODUCTION

The current guidelines recommend standard dual antiplatelet therapy (DAPT), that is, aspirin plus a P2Y12 inhibitor for at least 12 months in acute coronary syndrome (ACS) and for at least 6 months in stable coronary artery disease.^1^, ^2^ A standard dual antiplatelet regimen reduces the risk of ischemic events with an unacceptable increase in the risk of adverse events. The Clopidogrel versus Aspirin in Patients at Risk of Ischemic Events (CAPRIE) trial suggested that clopidogrel had a significant advantage compared with aspirin alone for the prevention of stroke, myocardial infarction (MI) in patients with atherosclerotic vascular disease.^3^ Meta‐analyses found that the short‐term DAPT (<12 months) duration was associated with similar risk of ischemic events and a lower risk of bleeding after percutaneous coronary intervention (PCI) than 12‐month duration.^4^ An observational study that compared clopidogrel monotherapy with aspirin monotherapy after 12‐month DAPT after drug eluting stent (DES) implantation, provides hypothesis‐generating evidence that clopidogrel monotherapy had more prognostic benefit than aspirin alone after DAPT in patients after percutaneous coronary intervention (PCI).^5^

P2Y12 inhibitor monotherapy was considered to be a new alternative antiplatelet strategy in patients after implantation of stents. Shortening the duration of aspirin therapy with more prolonged use of potent P2Y12 inhibitors may avoid aspirin‐related adverse risk. We aimed to explore the efficacy and safety of P2Y12 inhibitor monotherapy followed by short‐duration dual antiplatelet therapy in patients after PCI on the basis of the available evidence from all randomized controlled trials (RCTs).

## 2 METHODS

### 2.1 Data sources and searches

This systematic review was performed in accordance with the Preferred Reporting Items for Systematic Reviews (PRISMA) and meta‐analyses guidelines.^6^ A systematic search from PubMed, EMBASE (by Ovidsp), Web of Science, and The Cochrane Library was conducted from database inception to 2 October 2019, without a language restriction. Pre‐defined search key words included P2Y12 inhibitor monotherapy, dual antiplatelet therapy, percutaneous coronary intervention, and randomized controlled trial.

### 2.2 Study selection

Trials were selected if they (a) enrolled patients undergoing percutaneous coronary intervention, (b) were randomized clinical trials comparing P2Y12 antagonist monotherapy followed by short‐duration dual antiplatelet therapy with a dual antiplatelet regimen; and (c) reported clinically relevant efficacy and safety endpoints, such as cardiovascular events and bleeding endpoints.

### 2.3 Data extraction and quality assessment

After removal of duplicates, two investigators screened the search records by titles and abstracts. Disagreements were resolved through consensus. The same investigators independently recorded data on details of the included studies (ie, the study design, patients' characteristics, study and control treatment, follow‐up time, outcomes), and assessed the quality of the included randomized trials by the Cochrane collaboration's tool across five domains (sequence generation, allocation concealment, blinding of participants and researchers, outcomes assessment, and selection reporting).^7^ Differences were cross‐checked by a third reviewer.

### 2.4 Study outcomes

The primary efficacy outcome of interest was composite cardiovascular outcome (death, stroke, or MI). Secondary efficacy outcomes included all‐cause mortality, MI, stroke (included ischemic, hemorrhagic, and unknown), and stent thrombosis. Safety endpoint was the bleeding, which was individually defined across studies.

### 2.5 Data synthesis and analysis

This statistical analysis was performed using RevMan software (version 5.3). Fixed‐effects or random‐effects models were selected for each outcome based on the heterogeneity of the included trials, which was evaluated using the *I* ^2^ statistic.^8^ A random‐effects model was used for outcomes with high heterogeneity (*I* ^2^ > 50%). Pooled outcomes were presented as relative risks (RRs) and 95% confidence intervals (CIs).^9^ Differences between pooled RRs were assessed using *z* tests. Publication bias was not examined due to the small number of studies limiting the ability of funnel plots or regression analysis to test for bias.^10^ Subgroup analyses were conducted by the type of P2Y12 inhibitors.

## 3 RESULTS

**Figure 1:**
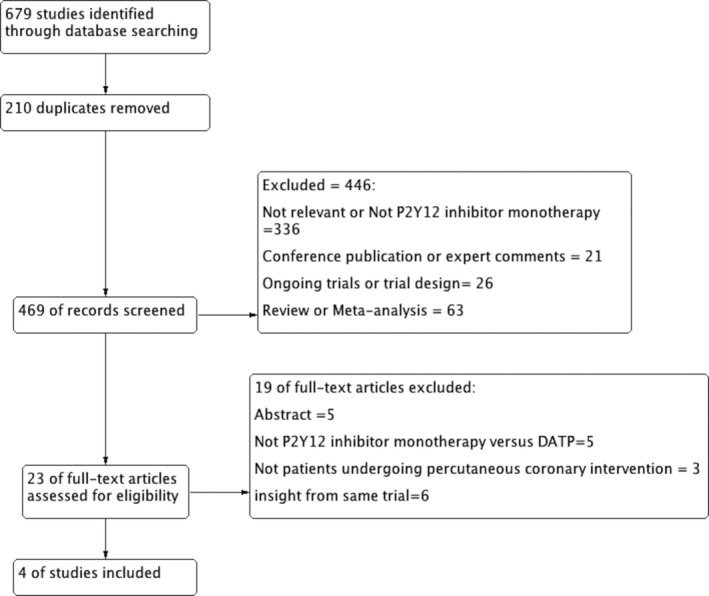
Flowchart for study selection

As described in Figure 1, the initial search identified 679 results. After removal of duplicates, 469 were screened based on inclusion criteria. At last, three open‐label and one double‐blind trials that included 29 089 patients met our eligibility criteria.^11^, ^12^, ^13^, ^14^ Of these, 14 530 patients were randomized to monotherapy with a P2Y12 inhibitor, whereas 14 599 patients were randomized to standard dual antiplatelet therapy. Four trials tested P2Y12 inhibitor monotherapy after 1 to 3 month of DAPT vs P2Y12 inhibitor plus aspirin (Table 1). The GLOBAL LEADERS trial^11^ defines composite cardiovascular outcome as a composite of all‐cause mortality or nonfatal centrally adjudicated new Q‐wave myocardial infarction (Table 1). The characteristics of patients in the included trials are shown in Table 2. Among patients eligible for the study, the mean age ranged from 53.7 to 79.5 years, the majority of patients were men, and more than 50% of participants had hypertension. The TWILIGHT study mainly includes high‐risk patients.

**Table 1:** Main characteristics of the studies included in meta‐analysis

Study	Year	Design	Patients	Intervention	Control	N	Follow‐up	End point
GLOBAL LEADERS	2018	RCT	PCI	ASA + TIC 1 mo → 23 mo TIC N = 7980	DAPT 12MON → ASA 12MON N = 7988	15 968	30 days,3, 6, 12, 18, 24 mo	A composite of all‐cause mortality or nonfatal centrally adjudicated new Q‐wave MI; stent thrombosis, bleeding
SMART‐CHOICE	2019	RCT	PCI	DAPT 3 mo → 9 mo P2Y12 N = 1495	DAPT 12MON N = 1498	2993	3，6，12 mo	A composite of all‐cause death, MI, or stroke；stent thrombosis, bleeding
STOPDAPT‐2	2019	RCT	PCI	DAPT 1 mo → 12 mo CLO N = 1500	DAPT 12MON(ASA + CLO) N = 1509	3045	12 mo	A composite of cardiovascular death, MI, ischemic or hemorrhagic stroke； stent thrombosis, bleeding
TWILIGHT	2019	RCT	PCI	DAPT 3 mo → 12 mo TIC	DAPT 3 Mon →DAPT 12MON	7119	18 mo	A composite of all‐cause death, MI, or stroke; stent thrombosis; bleeding

Abbreviations: ASA: aspirin; CLO: clopidogrel; DAPT: dual antiplatelet therapy; MI: myocardial infarction; PCI: percutaneous coronary intervention; RCT, randomized controlled trial; TIC: ticagrelor; ;

**Table 2:** Characteristics of patients in the included trials^a^

	GLOBAL LEADERS		SMART‐CHOICE		STOPDAPT‐2		TWILIGHT	
	Monotherapy	DAPT	Monotherapy	DAPT	Monotherapy	DAPT	Monotherapy	DAPT
Age, mean, y	64.5 ± 10.3	64.6 ± 10.3	64.6 ± 10.7	64.4 ± 10.7	68.1 ± 10.9	69.1 ± 10.4	65.2 ± 10.3	65.1 ± 10.4
Men	76.6	76.9	72.7	74.2	78.9	76.5	76.2	76.1
Hypertension	74.0	73.3	61.6	61.3	73.7	74.0	72.6	72.2
Hypercholesterolaemia	69.3	70.0	45.1	45.5	74.4	74.8	60.7	60.2
Prior myocardial infarction	23.0	23.6	4.1	4.3	13.8	13.2	28.7	28.6
Prior percutaneous coronary intervention	32.7	32.7	—	—	33.5	35.1	42.3	42.0
Previous stroke	2.6	2.6	6.6	6.8	5.4	7.0	20.4	23.1
Diabetes mellitus	25.7	24.9	38.2	36.8	39.0	38.0	37.1	36.5
Impaired renal function	13.9	13.5	2.9	3.5	5.5	5.6	16.8	16.7
Stable angina	53.0	53.2	41.8	41.8	62.3	61.4	29.5	28.0
Acute coronary syndrome								
Unstable angina	12.6	12.7	31.2	32.8	12.9	14.2	35.1	34.9
NonST‐segment elevation myocardial infarction	21.1	21.1	16.0	15.4	5.4	6.6	28.8	30.8
ST‐segment elevation myocardial infarction	13.3	12.9	11.0	10.0	19.4	17.9	—	—
Treated lesions								
Left main coronary artery	1.9	1.8	1.2	1.9	2.9	2.5	5.2	4.7
Left anterior descending artery	41.2	42.0	48.8	50.4	55.2	56.6	56.1	56.4
Left circumflex artery	24.3	24.5	21.6	19.9	17.9	20.2	32.4	32.2
Right coronary artery	31.6	30.7	28.3	27.8	29.1	27.2	35.0	35.3
Mean total stent length per lesion, mm (SD)	24.8 ± 13.9	24.8 ± 14.0	38.0 ± 22.5	27.8 ± 22.9	30.3 ± 16.7	30.5 ± 16.8	40.1 ± 24.2	39.7 ± 24.3

a Values are % or mean ± SD.

Abbreviation: DAPT, dual antiplatelet therapy.

Risk of bias assessment for each randomized controlled trial (RCT) was on the basis of Cochrane Collaboration guidelines. No evidence of high risk of bias was noted in three open‐label and one double‐blind study (Figure S1). Basically, no significant heterogeneity was found in the trial outcomes of these studies, with the exception of stroke (*I* ^2^ = 57%) and bleeding outcomes (*I* ^2^ = 73%). A random‐effects model was used for outcomes in this study.

**Figure 2:**
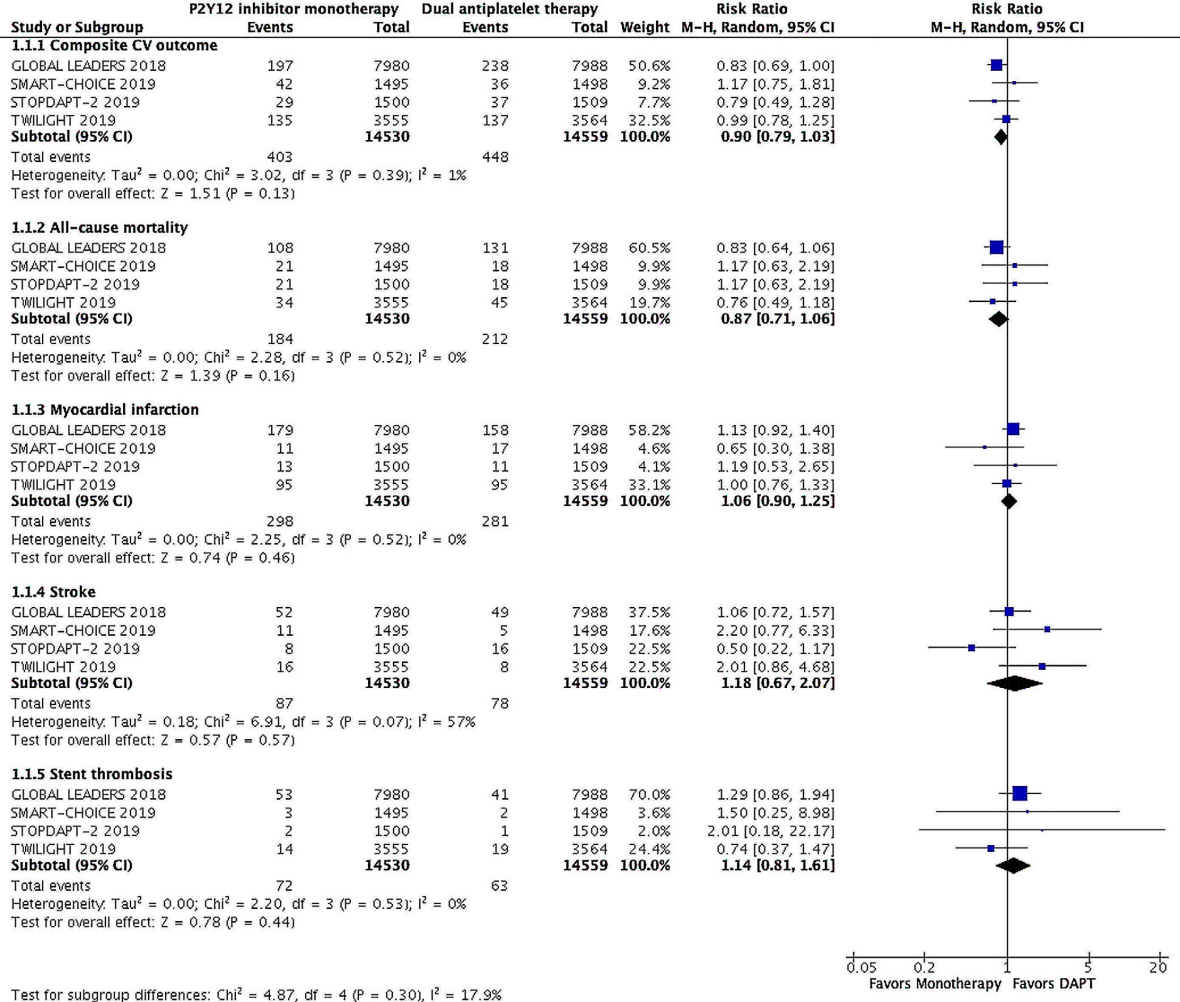
The effect of P2Y12 inhibitor monotherapy on cardiovascular outcome after PCI. 1.1.1 Composite cardiovascular outcome (CV outcome), 1.1.2 All‐cause mortality, 1.1.3 myocardial infarction (MI), 1.1.4 stroke, and 1.1.5 stent thrombosis. Squares represent the risk ratio of the individual studies. Horizontal lines represent the 95% confidence intervals (CIs) of the risk ratios (RR). The size of the square reflects the weight that the corresponding study contributes in the meta‐analysis. The diamond represents the pooled risk ratio or the overall effect

**Figure 3:**
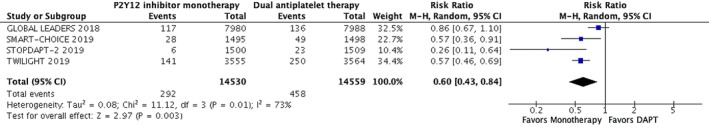
The effect of P2Y12 inhibitor monotherapy on the bleedings after PCI. Squares represent the risk ratio of the individual studies. Horizontal lines represent the 95% CIs of the risk ratios. The size of the square reflects the weight that the corresponding study contributes in the meta‐analysis. The diamond represents the pooled risk ratio or the overall effect

The pooled results for four trials are revealed in the forest plot. There was not associated with significant reductions in a composite endpoint of cardiovascular outcomes (2.8% vs 3.1%; RR: 0.90; 95% CI: 0.79‐1.03; *P* = .13) at 12 months after PCI. Furthermore, there were no significant differences in all‐cause mortality (1.3% vs 1.5%; RR: 0.87; 95% CI, 0.71‐1.06; *P* = .16), myocardial infarction (2.1% vs 1.9%; RR, 1.06; 95% CI, 0.90‐1.25; *P* = .46), stroke (0.6% vs 0.5%; RR, 1.18; 95% CI, 0.67‐2.07; *P* = .57), or stent thrombosis (0.5% vs 0.4%; RR, 1.14; 95% CI, 0.81‐1.61; *P* = .44) between the two groups (Figure 2). Monotherapy with P2Y12 antagonist after short‐duration dual antiplatelet therapy was associated with a 40% lower risk of major bleeding than P2Y12 inhibitor plus aspirin (RR: 0.60; 95% CI: 0.43 to 0.84; *P* = .005) (Figure 3). These data indicated that P2Y12 antagonist alone after shortening the duration of aspirin therapy had no significant increase in the occurrence of a composite endpoint of cardiovascular outcomes, and markedly decreased the risk of bleeding events than the DAPT group.

The subgroups analysis according to the type of P2Y12 inhibitors for the bleeding events, the date of clopidogrel alone is limited. The therapeutic effects of ticagrelor monotherapy compared with DAPT were consistent with pooled result (Figure S2). But it still needs to be further confirmed.

## 4 DISCUSSION

In this study, the efficacy and safety of P2Y12 inhibitor monotherapy followed by short‐duration dual antiplatelet therapy in patients after PCI on the basis of the available evidence from all randomized controlled trials was assessed. We found that P2Y12 antagonist alone after reducing the duration of dual antiplatelet therapy had no significant difference in the occurrence of a composite endpoint of cardiovascular outcomes compared with that in the DAPT group, but markedly decreased the risk of bleeding. The P2Y12 inhibitor monotherapy after short‐duration dual antiplatelet therapy might be noninferior to the current standard of care in terms of the reduced risk of bleeding events. In general, P2Y12 inhibitor monotherapy appears to reduce adverse events than P2Y12 inhibitor plus aspirin.

Platelet activation and aggregation play key roles in initiating and propagating coronary thrombosis. In the platelet inhibition and patient outcomes (PLATO) trial, ticagrelor, compared with clopidogrel, significantly reduced ischemic events and mortality without increasing the rate of overall major bleeding, but with increased the risk of minor bleeding.^15^ Reduction of bleeding after PCI is of great importance, because it has a strong relationship with subsequent all‐cause mortality and major adverse cardiovascular events.^16^ Furthermore, interruption of antiplatelet therapy because of bleeding may be associated with increased risk of thrombotic events. Optimal regimen of antiplatelet treatment in patients after PCI has been a topic of intense debate in recent years. Several studies have shown that prolonged dual antiplatelet therapy is associated with a trade‐off between ischemic and bleeding risks. In the dual antiplatelet therapy study, prolongation of treatment for an additional 18 months (beyond 1 year) significantly reduced major adverse cardiovascular events and stent thrombosis. However, all‐cause mortality and rates of moderate and severe bleeding increased.^17^ In previous studies, attempts to decrease the intensity of DAPT were mainly in patients with high risk of bleeding. It was reported that P2Y12 inhibitor monotherapy inhibited the activation of hemostatic system to a comparable extent compared with dual antiplatelet therapy.^18^ In this study, P2Y12 antagonist monotherapy was noninferior to dual antiplatelet therapy in clinical efficacy, but markedly reduced the risk of bleeding.

The findings of the SMART‐CHOICE trial and the STOPDAPT‐2 trial showed that monotherapy with a P2Y12 inhibitor after short‐duration dual antiplatelet therapy was noninferior to 12‐month DAPT in the Asian patients who were at lower risk for ischemic events.^12^, ^13^ There was no restriction on the type of P2Y12 inhibitors in the SMART‐CHOICE trial. On the contrary, the STOPDAPT‐2 trial is limited by the exclusive use of clopidogrel. Nevertheless, in the subgroup analysis of this study, there was no significant interaction between the type of P2Y12 inhibitors and the treatment effects of the two antiplatelet regimens on the bleeding events. The TWILIGHT study,^14^ enrolled patients undergoing PCI who are at high risk for ischemic or hemorrhagic complications, was associated with a 51% lower risk of bleeding. In contrast to these three studies, the results of The GLOBAL LEADERS trial showed that DAPT for 1 month followed by 23 months of ticagrelor alone did not reduce the incidence of bleeding than the traditional DAPT treatment regimen.^11^ Patients included in this analysis mainly were treated with ticagrelor alone after 1 to 3 month of P2Y12 inhibitors plus aspirin. These findings may be attributable to the differences in the research design, type of P2Y12 inhibitors, duration of therapy after randomization, and so on.

Overall, these trials with a large sample of evidence indicated that P2Y12 inhibitor monotherapy followed by very short‐duration dual antiplatelet therapy may be the future direction of antiplatelet therapy in patients after PCI.

## 5 LIMITATIONS

This study has several limitations. First, the inherent limitations of meta‐analyses exist, including quality and availability of the original data. Second, the sensitivity analysis was not evaluated due to the small number of trials. Third, the design and definitions of endpoints differed between those studies, which potentially reduced precision in the analyses. Fourth, the duration of DAPT after randomization was different in three eligible trials that might influence the risk of pooled endpoints, the TWILIGHT trial tested the results between randomization and 1 year, whereas the follow‐up endpoints of the other three trials in our analysis were 12 months after PCI. In addition, the included patients were from different countries, and the incidence of ischemic events might be relatively low in Asian participants compared with US and European patients. Sixth, the majority of patients had stable coronary heart disease. Except the TWILIGHT trial, other studies are mainly enrolled low‐risk patients after PCI. Therefore, it still needs further researches to prove that P2Y12 inhibitor monotherapy is appropriate to patients after PCI.

## 6 CONCLUSION

P2Y12 inhibitor monotherapy did not show any significant difference in the adverse cardiac and cerebrovascular events, but markedly decreased the risk of bleeding among patients after PCI versus dual antiplatelet therapy. However, as a result of the limited data, this conclusion should be further confirmed by more clinical trials.

## CONFLICT OF INTEREST

The authors declare no potential conflict of interests.

## Supporting information


**Figure S1** Risk of bias in the RCTs

**Figure S2** Subgroup analysis for the effect of P2Y12 inhibitor monotherapy on the bleedings after PCI. Squares represent the risk ratio of the individual studies. Horizontal lines represent the 95% CIs of the risk ratios. The size of the square reflects the weight that the corresponding study contributes in the meta‐analysis. The diamond represents the pooled risk ratio or the overall effect.
