# Supplementary figures and images for: The efficacy and safety of P2Y12 inhibitor monotherapy in patients after percutaneous coronary intervention
Source: Clin Cardiol. 2019 Nov 28;43(3):235–41. doi: 10.1002/clc.23305 (PMC7068072; doi:10.1002/clc.23305)

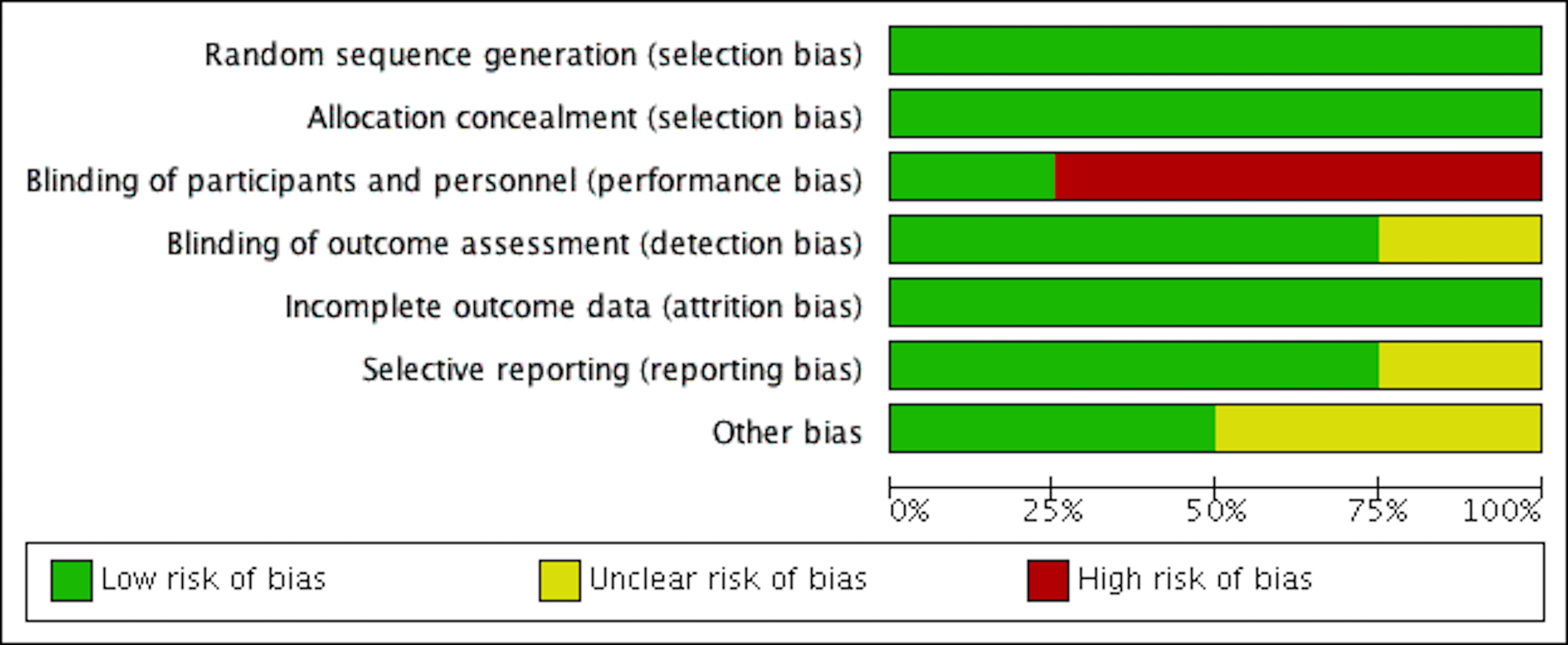

Supplement: Supplementary file 1 — Figure S1 Risk of bias in the RCTs [file CLC-43-235-s001.tiff]

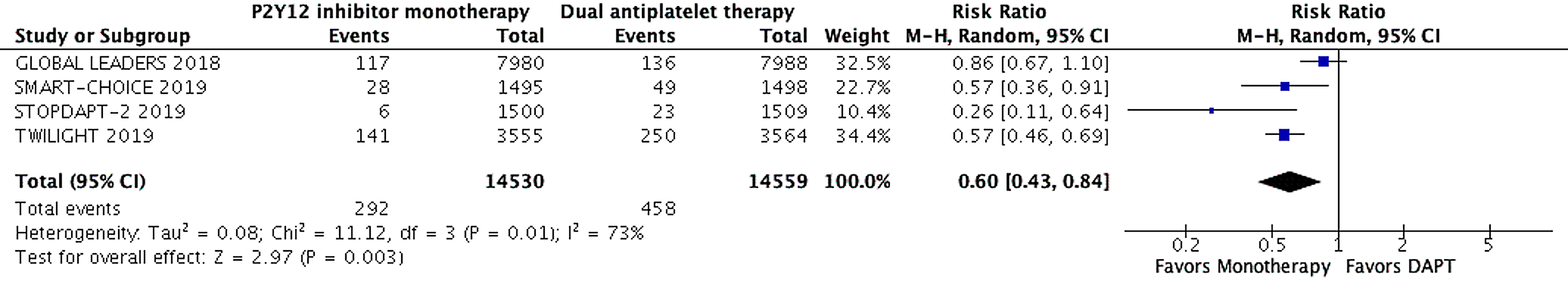

Supplement: Supplementary file 2 — Figure S2 Subgroup analysis for the effect of P2Y12 inhibitor monotherapy on the bleedings after PCI. Squares represent the risk ratio of the individual studies. Horizontal lines represent the 95% CIs of the risk ratios. The size of the square reflects the weight that the corresponding study contributes in the meta‐analysis. The diamond represents the pooled risk ratio or the overall effect. [file CLC-43-235-s002.tiff]
